# Military service experiences and reasons for service separation among lesbian, gay, and bisexual individuals in a large military cohort

**DOI:** 10.1186/s12889-021-12420-1

**Published:** 2022-01-06

**Authors:** Felicia R. Carey, Isabel G. Jacobson, Keren Lehavot, Cynthia A. LeardMann, Claire A. Kolaja, Valerie A. Stander, Rudolph P. Rull

**Affiliations:** 1 Deployment Health Research Department, Naval Health Research Center, 140 Sylvester Road, San Diego, CA 92106 USA; 2 Leidos, San Diego, CA USA; 3 Health Services Research & Development (HSR&D) Center of Innovation, VA Puget Sound Health Care System, Seattle, WA USA; 4 Department of Psychiatry & Behavioral Sciences, University of Washington, Seattle, WA USA; 5 Department of Health Services, University of Washington, Seattle, WA USA

**Keywords:** Military Service, Veterans, Millennium Cohort Study, Sexual Orientation, LGB Individuals

## Abstract

**Background:**

The well-being of lesbian, gay, and bisexual (LGB) individuals is a topic of increasing concern within the military where significant institutional barriers, targeted aggression, and differential organizational policies such as “Don’t Ask Don’t Tell” have historically contributed to experiences of exclusion and discrimination. However, limited research has examined specific military and post-separation experiences among LGB service members and veterans. The goal of this study was to examine differences in military and service separation experiences by sexual orientation among a large representative sample of United States service members and veterans.

**Methods:**

Survey data from the 2016 Millennium Cohort Study follow-up questionnaire were used to assess sexual orientation and multiple outcomes of interest: military experiences (morale, feelings about the military, missed workdays) and service separation experiences (reasons for separation, post-separation employment). The associations between sexual orientation (LGB versus heterosexual) and each of these outcomes were evaluated in a series of adjusted logistic regression models, stratified by sex when interactions were observed.

**Results:**

Of the 99,599 participants, 3.4% identified as LGB. In adjusted models, LGB service members had significantly higher odds than heterosexual service members of feeling: unimpressed by the quality of unit leadership, unsupported by the military, and negative about the military overall. Bisexual women were more likely than heterosexual women to feel less unit camaraderie; both gay and bisexual men felt less camaraderie than heterosexual men. LGB veterans were more likely than heterosexual peers of the same sex to separate from service due to unplanned administrative reasons. Compared to heterosexual women, lesbian and bisexual women were more likely to separate from service due to dissatisfaction with promotions/pay and disability/medical reasons, while bisexual women specifically separated due to dissatisfaction with leadership and incompatibility with the military. Gay and bisexual men also reported separating due to incompatibility with the military, but only bisexual men were more likely to report separating due to disability/medical reasons compared to heterosexual men.

**Conclusions:**

Less positive military- and separation-specific experiences disproportionately affected LGB service members in this study. Promoting inclusion and increasing support for LGB service members may improve satisfaction with military service and retention.

## Background

Lesbian, gay, and bisexual (LGB) individuals have a long-standing presence within military settings that simultaneously includes formative contributions to military and queer culture, as well as a complex history of exclusion and discharge from military service due to their sexual identities [1–5]. In more recent history, the institution of the 1993 Department of Defense Directive 1304.26, commonly referred to as “Don’t Ask, Don’t Tell” (DADT), allowed LGB individuals to serve as long as they concealed their sexual orientation [6]. It was not until the 2011 enactment of the DADT Repeal Act that LGB individuals were able to openly serve. Across this history, LGB personnel serving in the military have faced significant minority stress, defined as specific stressors such as discrimination and stigma that minorities face that are in excess of general stressors [7], overt or covert targeted aggression (e.g., discrimination, harassment, sexual assault) due to their sexual orientation [8–12], and differential organizational policies such as DADT that may further exacerbate alienation and discrimination [13]. Importantly, feelings of stress and anxiety have been reported by LGB military personnel as a result of DADT specifically [14]. The legacy of exclusion and discrimination against LGB individuals in the military setting may impede the development of trust and familiarity with fellow service members [15], and may also contribute to poorer psychological and physical health among LGB service members [8, 16–20].

While existing research has found that LGB individuals who have served in the military are more likely to report experiencing discrimination, harassment, and poor mental and physical health compared to heterosexual individuals, these studies were limited by their use of convenience samples [18–21] and most were comprised only of veterans, not current service members [8, 17–19, 22–24]. Furthermore, it is possible that service members identifying as LGB may have a higher risk of post-service unemployment based on findings from previous research showing associations of LGB identification with feelings of anxiety or stress [14], and associations between anxiety and depression and post-service unemployment [25]. To our knowledge, previous research has not assessed how military experiences, such as morale and unit cohesion, and service separation experiences, such as post-separation employment, may differ by sexual orientation.

The present study is one of the first to examine sexual orientation in relation to military and service separation experiences among a large longitudinal study of service members in the United States (US). Specifically, the main aim of this exploratory study was to examine whether military and service separation experiences differed among service members and veterans who identified as LGB, compared with those who identified as heterosexual. Thus, this study was designed to test the hypothesis that service members and veterans who identify as LGB have significantly higher levels of adverse military and post-separation experiences compared with those who identify as heterosexual.

## Methods

### Study population

The Millennium Cohort Study was designed to follow individuals through their military careers and after separation from service [26]. Individuals in the recruitment sampling frame were randomly selected from active duty, National Guard, and Reserve US military personnel rosters to represent all service components and branches and were recruited via mailings and e-mail invitations (see Ryan et al. [26] and Smith et al. [27] for detailed methodology). To ensure sufficient power to detect differences in smaller subgroups, specific demographics (e.g., women, Marines) were oversampled. The study is unable to oversample for specific sexual orientation groups, since this information is not available in personnel rosters. The Cohort currently includes nearly 250,000 participants enrolled during 5 separate cycles, with baseline enrollment between 2001 and 2019. Health outcomes and behaviors, occupational factors, and life experiences are assessed at the time of enrollment and every 3 to 5 years post-baseline using an in-depth questionnaire. The sample for the present study (*N* = 103,245) was drawn from participants from the first 4 enrollment panels who completed the full version of the 2014–2016 (2016) follow-up survey, which was the first time sexual orientation was assessed. Individuals who did not report their sexual orientation (*n* = 3644) or responded with “prefer not to answer” (*n* = 2671) were excluded from all analyses because of the heterogeneity of individuals in these groups and the inability to draw any conclusions regarding their sexual orientation. Therefore, the analytic sample consisted of 96,930 individuals. As of the 2016 survey, 56,645 (58.4%) of the analytic sample were veterans (i.e., any individual who has served in the military and is now separated from service) and were included in analyses of separation experiences.

### Measures

#### Sexual orientation

Sexual orientation was measured by a single item asking, “Do you consider yourself to be …” , with response options including “heterosexual or straight”, “gay or lesbian”, or “bisexual”. As noted, individuals who endorsed the fourth response option “prefer not to answer” were excluded from analyses.

#### Military experiences

Three morale-related items, modified from the Deployment Risk and Resilience Inventory [28], assessed agreement on a 5-point Likert scale. Based on the most recent duty assignment, the items were: 1) “I was impressed by the quality of leadership in my unit”, 2) “I felt a sense of camaraderie between myself and others in my unit”, and 3) “I was supported by the military”. Response options were collapsed into 3 levels (agree, neither agree nor disagree, and disagree). A single item, “What is your overall feeling about your military service?”, assessed attitudes toward the military with response options collapsed into 3 levels (negative, neither negative nor positive, and positive). Self-reported number of days in the past 3 years participants had missed work due to illness or injury was categorized as 0 to 2 days, 3 to 7 days, 8 to 21 days, and 22+ days.

#### Service separation experiences

Self-reported reasons for service separation were assessed by asking, “What was the reason for your separation/retirement from the military?”; response options included “planned separation”, “medical separation”, “disciplinary separation”, “unplanned administrative separation (e.g., military downsizing, failure to promote, failure to meet service standards)”, and “other (e.g., pregnancy, parenthood, educational pursuits)”. Missing responses (5%) were backfilled using interservice separation codes for corresponding categories obtained from Defense Manpower Data Center (DMDC) personnel records.

To further explore reasons for separation from service, participants rated 11 items on a 5-point scale (from “not at all” to “extremely”) that asked, “How much did each of the following reasons affect your decision to leave the military?” These items have been examined in Millennium Cohort Study subpopulations previously [29] and included: 1) “dissatisfaction with deployments and/or frequent moves”, 2) “military service created hardship for family”, 3) “dissatisfaction with promotion, pay, or other benefits”, 4) “dissatisfaction with job”, 5) “dissatisfaction with leadership/supervision”, 6) “desire to continue your education, start a new career, or change in personal goals”, 7) “disability or other medical reasons”, 8) “difficulty meeting weight standards and/or fitness standards”, 9) “incompatibility with the military”, 10) “legal problems or problems meeting a military obligation”, and 11) “fulfilled term of service or was retirement eligible”. Responses to each item were dichotomized as “not at all” versus “any agreement”.

Post-separation employment status was self-reported and categorized as “full-time, part-time”, “not employed, looking for work”, “retired or not looking for work”, “not employed, disabled”, and “homemaker or other”.

#### Covariates

Demographics including sex, birth year, and race/ethnicity, were obtained from DMDC records at the time of baseline enrollment. Education level, marital status, and enrollment panel were self-reported on the 2016 survey and backfilled using DMDC records if missing.

Military characteristics, including service status, service branch, pay grade, length of military service, and military occupation, were obtained from DMDC records using the most recent record available corresponding to the 2016 survey date. Because deployment and combat exposure significantly predict mental and behavioral health sequelae influencing retention and resilience [30], deployment with combat experience was assessed using data from the Contingency Tracking System in combination with survey data. Those who had an electronic record of a deployment prior to the 2016 survey were categorized as ever deployed. Individuals who had deployed were further categorized based on whether they self-reported 1 or more combat experiences (e.g., witnessing a person’s death due to war, being attacked or ambushed, being wounded or injured) on any survey between baseline and the 2016 survey [30]. Deployment experience was categorized as “never deployed”, “deployed with no combat exposure”, and “deployed with combat exposure”.

### Statistical analyses

Multiple imputation with 10 imputations [31] was used to backfill missing responses on all demographic and military characteristics, with the percentage missing prior to imputation among the analytic sample ranging from <0.01 to 0.05%. Descriptive frequencies of military experiences, service separation experiences, and demographic and military characteristics were reported by sexual orientation. Associations between sexual orientation and each military and service separation experience outcome were estimated using adjusted odds ratios (AORs) derived from separate logistic regression models that included demographic and military characteristics and study panel as covariates. Service separation outcomes were modeled among veteran participants only. Since women serving in the military may experience specific psychosocial and interpersonal stress [32–34], interactions between sex and sexual orientation were examined for all models to determine whether effect estimates differed by sex. For associations that were modified by sex (*p*-value<.05), models stratified by sex are presented. Interactions between service status and sexual orientation were also examined for military experiences models given potential differences between current and former service members; significant differences by service status (interaction *p*-value<.05) are described in text only.

## Results

### Descriptive frequencies

Among 96,930 eligible participants, 96.4% of the population identified as heterosexual, 1.9% as gay or lesbian, and 1.7% as bisexual (Table 1). Consistent across sexual orientations, the largest proportion of individuals were non-Hispanic white (69.7–76.6%), born in 1980 or later (41.0–62.3%), veterans (58.3–63.7%), in the Army (44.6–49.6%), of a senior enlisted pay grade (65.9–74.0%), in a military occupation other than combat specialist, functional support/ administration, or health care (50.8–54.9%), and had deployed with combat exposure (44.7–48.2%). The majority of service members who identified as lesbian/gay (64.0%) and bisexual (59.6%) were female, whereas most of those who identified as heterosexual (72.0%) were male. Also, most service members who identified as heterosexual (71.5%) reported being married or in a committed relationship, while the majority of LGB individuals reported being either single or separated, divorced, or widowed (51.2–68.9%).

**Table 1:** Sociodemographic characteristics by sexual orientation, millennium cohort study, 2016 (*N* = 96,930)

Variable	Sexual Orientation [n (column %)]		
	Heterosexual (*n* = 93,492; 96.4%)	Gay or Lesbian (*n* = 1824; 1.9%)	Bisexual (*n* = 1614; 1.7%)
**Demographic Characteristics**			
Sex			
Male	67,305 (72.0)	656 (36.0)	652 (40.4)
Female	26,187 (28.0)	1168 (64.0)	962 (59.6)
Birth year			
Pre-1960	12,168 (13.0)	156 (8.6)	60 (3.7)
1960–1969	19,677 (21.1)	353 (19.4)	160 (9.9)
1970–1979	23,275 (24.9)	425 (23.3)	389 (24.1)
1980+	38,372 (41.0)	890 (48.8)	1005 (62.3)
Race and ethnicity*			
Black, non-Hispanic	9316 (10.0)	214 (11.7)	144 (8.9)
Hispanic	6517 (7.0)	179 (9.8)	145 (9.0)
Other^a^	6008 (6.4)	160 (8.8)	120 (7.4)
White, non-Hispanic	71,649 (76.6)	1271 (69.7)	1205 (74.7)
Marital status			
Single or dating casually	11,683 (12.5)	929 (50.9)	379 (23.5)
Married or in a committed relationship	66,796 (71.5)	567 (31.1)	788 (48.8)
Separated, divorced, or widowed	15,013 (16.1)	328 (18.0)	447 (27.7)
Education level			
High school or less	5646 (6.0)	63 (3.5)	89 (5.5)
Some college or associate degree	39,424 (42.2)	778 (42.7)	876 (54.3)
Bachelor’s degree	25,083 (26.8)	508 (27.9)	387 (24.0)
Master’s degree or higher	23,339 (25.0)	475 (26.0)	262 (16.2)
**Military Characteristics**			
Service status			
Active duty	23,498 (25.1)	350 (19.2)	350 (21.7)
Reserve/National Guard	15,526 (16.6)	325 (17.8)	236 (14.6)
Veteran	54,468 (58.3)	1149 (63.0)	1028 (63.7)
Service branch			
Army	41,648 (44.6)	855 (46.9)	800 (49.6)
Navy/Coast Guard	16,956 (18.1)	407 (22.3)	326 (20.2)
Marine Corps	7036 (7.5)	77 (4.2)	108 (6.7)
Air Force	27,852 (29.8)	485 (26.6)	380 (23.5)
Pay grade*			
Junior enlisted	5020 (5.4)	171 (9.4)	207 (12.8)
Senior enlisted	61,574 (65.9)	1227 (67.3)	1194 (74.0)
Officer	26,897 (28.8)	426 (23.4)	213 (13.2)
Military occupation*			
Combat Specialist	16,266 (17.4)	142 (7.8)	156 (9.7)
Functional Support/ Administration	17,871 (19.1)	423 (23.2)	326 (20.2)
Health care	11,934 (12.8)	304 (16.7)	246 (15.3)
Other	47,459 (50.8)	955 (52.4)	886 (54.9)
Length of Military Service^b^			
Years, Mean (SD)	12.7 (8.8)	10.6 (7.7)	8.3 (6.8)
Deployment experience*			
Never deployed	34,798 (37.2)	740 (40.6)	662 (41.0)
Deployed, no combat	13,586 (14.5)	242 (13.3)	230 (14.3)
Deployed, with combat	45,061 (48.2)	841 (46.1)	722 (44.7)

Note. Asterisks (*) indicate that the distribution of the specified covariate is presented prior to multiple imputation. Sample sizes for these items range from 96,882 to 96,929. Adjusted models utilize the imputed versions of these covariates

^a^Including American Indian (*n* = 1196), Asian and Pacific Islander (*n* = 3896), and Other racial and ethnic groups (e.g., multiple races and ethnicities, *n* = 1196) as identified within Defense Manpower Data Center personnel records

^b^Length of military service was measured as time from each participant’s basic active service date to their date of separation, for veterans, or the 2016 survey date, for current service members

Compared with heterosexual service members (Table 2), higher proportions of LGB service members disagreed that they were impressed by the quality of leadership in their units, felt a sense of unit camaraderie, or felt supported by the military. Higher proportions of LGB service members also reported negative feelings about the military and missing 22+ days of work compared with heterosexual service members. While the majority of veterans had a planned separation, a greater proportion of LGB veterans reported unplanned separations compared with heterosexual veterans. The most commonly endorsed specific reason for separation from service among veterans who identified as heterosexual or lesbian/gay was fulfilling the term of service or being retirement eligible, while veterans who identified as bisexual most frequently endorsed dissatisfaction with leadership. A higher proportion of veterans who identified as heterosexual and lesbian/gay reported being employed full-time post-separation compared with veterans who identified as bisexual.

**Table 2:** Military and service separation experiences by sexual orientation, millennium cohort study, 2016

Outcome Variable	Sexual Orientation [n (column %)]		
	Heterosexual	Gay or Lesbian	Bisexual
**Military Experiences (*N*** **= 96,930)**			
Impressed by quality of leadership			
Agree	45,342 (50.0)	744 (42.3)	586 (37.6)
Neither agree nor disagree	19,480 (21.5)	393 (22.4)	347 (22.2)
Disagree	25,839 (28.5)	620 (35.3)	627 (40.2)
Felt a sense of unit camaraderie			
Agree	68,710 (75.8)	1200 (68.3)	973 (62.5)
Neither agree nor disagree	10,614 (11.7)	254 (14.5)	232 (14.9)
Disagree	11,360 (12.5)	304 (17.3)	353 (22.7)
Felt supported by the military			
Agree	58,524 (64.6)	956 (54.4)	757 (48.5)
Neither agree nor disagree	17,970 (19.8)	389 (22.2)	375 (24.0)
Disagree	14,171 (15.6)	411 (23.4)	428 (27.4)
Feelings about the military			
Positive	76,682 (84.5)	1373 (78.2)	1116 (71.5)
Neutral	7156 (7.9)	177 (10.1)	191 (12.2)
Negative	6964 (7.7)	205 (11.7)	255 (16.3)
Missed days of work (last 3 years)			
0 to 2	47,035 (52.7)	788 (45.5)	663 (43.4)
3 to 7	13,527 (15.2)	280 (16.2)	203 (13.3)
8 to 21	13,624 (15.3)	282 (16.3)	291 (19.0)
22+	15,024 (16.8)	382 (22.1)	371 (24.3)
**Service Separation Experiences among Veterans (*N*** **= 56,645)**			
Reason for military separation			
Planned separation	42,823 (78.6)	797 (69.4)	623 (60.6)
Medical separation	5421 (10.0)	164 (14.3)	177 (17.2)
Disciplinary separation	741 (1.4)	26 (2.3)	37 (3.6)
Unplanned administrative separation	2596 (4.8)	101 (8.8)	85 (8.3)
Other	2887 (5.3)	61 (5.3)	106 (10.3)
Specific reasons for separation (any endorsement)^a^			
Dissatisfaction with deployments/frequent moves	18,637 (36.0)	354 (32.9)	373 (38.4)
Hardship for family	22,738 (43.9)	344 (32.0)	452 (46.5)
Dissatisfaction with promotion, pay, benefits	17,910 (34.6)	406 (37.9)	397 (41.0)
Dissatisfaction with job	17,230 (33.3)	420 (39.2)	427 (44.1)
Dissatisfaction with leadership	26,039 (50.3)	596 (55.7)	590 (60.8)
Desire to continue education, start new career, change in personal goals	25,109 (48.5)	551 (51.4)	545 (56.2)
Disability or medical reasons	15,672 (30.3)	387 (36.2)	433 (44.5)
Difficulty meeting fitness/weight standards	11,584 (22.4)	289 (27.0)	303 (31.1)
Incompatibility with military	5767 (11.2)	221 (20.6)	237 (24.4)
Legal problems, problems meeting military obligation	1520 (2.9)	43 (4.0)	65 (6.7)
Fulfilled term of service/ retirement eligible	35,550 (68.7)	638 (59.6)	532 (54.9)
Post-separation employment status			
Full-time	36,125 (66.8)	738 (64.5)	570 (55.8)
Part-time	3715 (6.9)	90 (7.9)	107 (10.5)
Not employed, looking for work	2800 (5.2)	84 (7.3)	95 (9.3)
Retired or not looking for work	5062 (9.4)	96 (8.4)	56 (5.5)
Not employed, disabled	2483 (4.6)	66 (5.8)	79 (7.7)
Homemaker or other	3924 (7.3)	71 (6.2)	115 (11.3)

Note. Separation experiences are assessed among the veteran population only. Base sample sizes are as noted. Due to nonresponse, the number of missing responses on each outcome variable vary. For military experiences items, the analytic sample size ranges from 92,470 to 94,119. For service separation experiences, the analytic sample size ranges from 53,709 to 56,645

^a^Column percentages for specific reasons for separation do not sum to 100%. Each item represents a separate survey question. Presented sample sizes and percentages represent the proportion of individuals who reported any endorsement of each specific reason

### Adjusted analyses

Adjusted odds ratios for military experiences by sexual orientation, not modified by sex, are shown in Table 3. After adjustment for demographic and military characteristics, LGB service members were more likely than heterosexual service members to disagree that they were impressed by the quality of leadership in their units and disagree that they felt supported by the military. LGB service members were also significantly more likely than heterosexual service members to report negative feelings about the military.

**Table 3:** Adjusted odds ratios of military experiences by sexual orientation (*N* = 96,930)

Outcome Variable	Sexual Orientation (Ref: Heterosexual)	
	Gay or Lesbian	Bisexual
	OR (95% CI)	OR (95% CI)
**Impressed by quality of leadership** (Ref: agree)		
Neither agree nor disagree	1.01 (0.89, 1.15)	1.03 (0.90, 1.18)
Disagree	**1.14 (1.01, 1.27)**	**1.24 (1.10, 1.40)**
**Felt supported by the military** (Ref: agree)		
Neither agree nor disagree	1.07 (0.95, 1.21)	**1.16 (1.02, 1.32)**
Disagree	**1.36 (1.21, 1.54)**	**1.51 (1.33, 1.71)**
**Feelings about the military** (Ref: positive)		
Neither negative nor positive	1.07 (0.91, 1.26)	**1.25 (1.06, 1.46)**
Negative	**1.23 (1.05, 1.43)**	**1.57 (1.36, 1.81)**

Note. No significant effect modification by sex was observed for these models. All models are adjusted for sex, birth year, race/ethnicity, marital status, education level, service branch, service status, pay grade, military occupation, length of service, deployment experience, and panel. Bolded values are statistically significant (*p* < 0.05)

*CI* confidence interval, *OR* odds ratio

Adjusted odds ratios, stratified by sex, are shown in Table 4, where the associations of sexual orientation with military experiences were significantly modified by sex (interaction *p*-value<.05). After adjustment for demographic and military characteristics, only bisexual women had higher odds than heterosexual women of disagreeing that they felt a sense of unit camaraderie, while both gay and bisexual men had higher odds than heterosexual men of disagreeing that they felt a sense of unit camaraderie. All LGB service members were more likely than their heterosexual same-sex peers to miss 3 or more days of work in the past 3 years. However, both lesbian and bisexual women were more likely to miss 22+ days compared with heterosexual women, while only bisexual men were more likely than heterosexual men to miss 22+ days.

**Table 4:** Adjusted odds ratios of military experiences by sex and sexual orientation (*N* = 96,930)

Outcome Variable	Female		Male	
	Sexual Orientation (Ref: Heterosexual)		Sexual Orientation (Ref: Heterosexual)	
	Lesbian	Bisexual	Gay	Bisexual
	OR (95% CI)	OR (95% CI)	OR (95% CI)	OR (95% CI)
**Felt a sense of unit camaraderie** (Ref: agree)				
Neither agree nor disagree	0.98 (0.82-1.17)	1.19 (0.99-1.43)	**1.30 (1.04-1.64)**	1.08 (0.84-1.39)
Disagree	1.12 (0.95-1.32)	**1.37 (1.17-1.61)**	**1.34 (1.07-1.68)**	**1.84 (1.51-2.25)**
**Missed days of work in last 3 years** (Ref: 0 to 2 days)				
3 to 7 days	**1.39 (1.17-1.66)**	1.05 (0.84-1.30)	1.11 (0.88-1.40)	1.17 (0.93-1.49)
8 to 21 days	1.16 (0.97-1.39)	**1.31 (1.09-1.58)**	**1.31 (1.05-1.64)**	**1.57 (1.27-1.94)**
22+ days	**1.59 (1.36-1.86)**	**1.55 (1.31-1.84)**	1.14 (0.91-1.44)	**1.35 (1.09-1.68)**

Note. Significant effect modification by sex (interaction *p* < 0.05) was observed for these models. All models are adjusted for birth year, race/ethnicity, marital status, education level, service branch, service status, pay grade, military occupation, length of service, deployment experience, and panel. Bolded values are statistically significant (*p* < 0.05)

*CI* confidence interval, *OR* odds ratio

The observed associations of sexual orientation with military experiences did not differ by service status (results not shown) with one exception. The association between sexual orientation and military support was modified by service status (interaction *p*-value = .032), such that bisexual active duty service members (adjusted odds ratio [AOR] = 1.70, 95% confidence interval [CI]:1.30-2.24) and veterans (AOR = 1.53, 95% CI:1.32-1.78) and lesbian and gay veterans (AOR = 1.58, 95% CI:1.37-1.83) had higher odds of disagreeing that they felt supported by the military compared to heterosexual peers of the same service status. There were no significant differences for military support between lesbian and gay active duty service members or LGB Reserve/National Guard service members compared to heterosexual service members of the same service status.

Adjusted odds ratios for service separation experiences among veterans by sexual orientation, not modified by sex, are shown in Table 5. After adjustment for demographic and military characteristics, bisexual individuals were more likely than heterosexual individuals to report that their service separation was due to dissatisfaction with the job, difficulty meeting fitness/weight standards, or legal problems/problems meeting military obligations. Gay or lesbian veterans were less likely than heterosexual veterans to report that their separation was due to family hardships or fulfillment of the term of service/retirement.

**Table 5:** Adjusted odds ratios of service separation experiences among veterans by sexual orientation (*N* = 56,645)

Outcome Variable	Sexual Orientation (Ref: Heterosexual)	
	Gay or Lesbian	Bisexual
	OR (95% CI)	OR (95% CI)
**Specific reasons for separation** (Ref: no endorsement)^a^		
Dissatisfaction with deployments/frequent moves	0.91 (0.80, 1.05)	1.07 (0.94, 1.23)
Hardship for family	**0.76 (0.66, 0.87)**	1.01 (0.88, 1.15)
Dissatisfaction with job	1.03 (0.90, 1.17)	**1.20 (1.05, 1.36)**
Desire to continue education, start new career, change in personal goals	0.90 (0.79, 1.03)	0.97 (0.84, 1.11)
Difficulty meeting fitness/weight standards	1.09 (0.95, 1.26)	**1.26 (1.09, 1.45)**
Legal problems, problems meeting military obligation	1.09 (0.79, 1.50)	**1.58 (1.21, 2.06)**
Fulfilled term of service/retirement eligible	**0.78 (0.68, 0.89)**	0.88 (0.77, 1.01)

Note. No significant effect modification by sex was observed for these models. All models are adjusted for sex, birth year, race/ethnicity, marital status, education level, service branch, pay grade, military occupation, length of service, deployment experience, and panel. Bolded values are statistically significant (*p* < 0.05)

*CI* confidence interval, *OR* odds ratio

^a^ORs represent the odds of any endorsement of each specific reason for separation versus no endorsement

Adjusted odds ratios, stratified by sex, are shown in Table 6, where the associations of sexual orientation with service separation experiences among veterans were significantly modified by sex (interaction *p*-value<.05). After adjustment for demographic and military characteristics, all LGB veterans had significantly higher odds than heterosexual veterans of the same sex of having an unplanned administrative separation. Compared to heterosexual veterans of the same sex, LGB veterans were also more likely to report a medical separation (lesbian women, gay and bisexual men), a disciplinary separation (bisexual women, gay men), and a separation for other reasons (gay men). LGB women were more likely to report more specific reasons for separation than heterosexual women (e.g., lesbian and bisexual women reported dissatisfaction with promotions/pay and disability/medical reasons, bisexual women reported dissatisfaction with leadership and incompatibility with the military). Gay and bisexual men also had higher odds than heterosexual men of reporting incompatibility with the military as a reason for separation, but only bisexual men had higher odds of reporting a separation due to disability/medical reasons. Compared with heterosexual veterans of the same sex, LGB veterans had higher odds of being unemployed due to disability (bisexual women and men) and unemployed but looking for work (bisexual men).

**Table 6:** Adjusted odds ratios of service separation experiences among veterans by sex and sexual orientation (*N* = 56,645)

Outcome Variable	Female		Male	
	Sexual Orientation (Ref: Heterosexual)		Sexual Orientation (Ref: Heterosexual)	
	Lesbian	Bisexual	Gay	Bisexual
	OR (95% CI)	OR (95% CI)	OR (95% CI)	OR (95% CI)
**Reason for separation** (Ref: planned separation)				
Disciplinary separation	1.46 (0.76-2.80)	**2.16 (1.30-3.58)**	**1.78 (1.01-3.14)**	1.65 (0.95-2.87)
Medical separation	**1.41 (1.12-1.77)**	1.22 (0.96-1.54)	**1.46 (1.05-2.02)**	**1.66 (1.25-2.22)**
Unplanned administrative separation	**1.60 (1.16-2.20)**	**1.47 (1.06-2.04)**	**1.90 (1.35-2.67)**	**1.84 (1.28-2.66)**
Other reason	**0.58 (0.41-0.82)**	1.11 (0.87-1.43)	**1.87 (1.13-3.10)**	1.60 (0.94-2.73)
**Specific reasons for separation** (Ref: no endorsement)^a^				
Dissatisfaction with promotion, pay, benefits	**1.28 (1.08-1.51)**	**1.35 (1.13-1.60)**	1.10 (0.90-1.35)	0.99 (0.81-1.22)
Dissatisfaction with leadership	1.14 (0.97-1.34)	**1.20 (1.01-1.43)**	0.87 (0.71-1.08)	1.01 (0.82-1.24)
Disability or medical reasons	**1.42 (1.20-1.67)**	**1.51 (1.27-1.79)**	1.00 (0.79-1.26)	**1.29 (1.04-1.59)**
Incompatibility with military	1.12 (0.90-1.39)	**1.53 (1.26-1.86)**	**2.06 (1.63-2.60)**	**1.75 (1.37-2.25)**
**Post-separation employment** (Ref: full-time)				
Part-time	**0.57 (0.43-0.77)**	1.19 (0.92-1.55)	1.37 (0.95-1.97)	1.39 (0.95-2.03)
Not employed, looking for work	0.80 (0.58-1.10)	1.32 (0.98-1.79)	1.30 (0.91-1.84)	**1.64 (1.16-2.31)**
Retired or not looking for work	0.81 (0.61-1.07)	1.05 (0.72-1.54)	1.27 (0.83-1.93)	1.16 (0.74-1.81)
Not employed, disabled	1.20 (0.87-1.65)	**1.46 (1.05-2.03)**	1.33 (0.84-2.11)	**1.81 (1.25-2.63)**
Homemaker or other	**0.48 (0.36-0.65)**	0.92 (0.73-1.18)	1.32 (0.81-2.14)	1.18 (0.74-1.88)

Note. Significant effect modification by sex (interaction *p* < 0.05) was observed for these models. All models are adjusted for birth year, race/ethnicity, marital status, education level, service branch, pay grade, military occupation, length of service, deployment experience, and panel. Bolded values are statistically significant (*p* < 0.05)

*CI* confidence interval, *OR* odds ratio

^a^ORs represent the odds of any endorsement of each specific reason for separation versus no endorsement

## Discussion

In this investigation of sexual orientation among nearly 97,000 participants from the Millennium Cohort Study, most of the participants had positive feelings about the military. However, LGB service members were more likely to report negative feelings about their military service compared with heterosexual individuals. LGB service members were also more likely to report being unimpressed by the quality of leadership in their units and feeling less supported by the military. Bisexual women and gay and bisexual men reported feeling less camaraderie with others in their units and separating due to incompatibility with the military more often than heterosexual peers of the same sex. These results are not entirely surprising given that “homosexuality” was previously a reason for dishonorable discharge from the military [35]. As such, findings from this study may suggest a need for equitable support for LGB service members and veterans to ensure that they are provided the same opportunities, by the military as a whole and at the unit level, as heterosexual service members. While recent Executive Orders (Exec. Order No. 13988, 2021) and Department of Defense Instructions (DoD Instruction 1350.02, DoD Instruction 1020.05) have provided guidance on the establishment of equal opportunity and diversity and inclusion programs in the Department of Defense, implementation of evidence-based strategies for addressing systemic social and health inequities among marginalized groups in the military are likely needed to improve the experiences of LGB service members. In addition, programs providing cultural competency training for leadership and health providers may positively impact the military and health care experiences of LGB service members, thereby improving retention among these individuals.

Findings suggested that LGB individuals may be more vulnerable to attrition before service term fulfillment than heterosexual individuals, given that LGB veterans were more likely to report unplanned administrative or medical separations and perceived incompatibility with the military as reasons for separation. A 2010 Congressional report showed that a total of 32,478 administrative separations for “homosexual conduct” occurred between 1980 and 2009; however, these individuals accounted for less than 0.1% of the total active force serving during this time period [6]. While the repeal of DADT eliminated administrative separations due to sexual orientation, other forms of administrative separation (e.g., failure to meet standards, character and behavior disorders) were observed as causes of premature separation among LGB individuals in the current study sample. Non-routine separations are associated with psychological and behavioral health issues post-separation that may have been preexisting and worsened by stressors occurring during military service [36]. This elevated frequency of non-routine separation may underscore existing disparities in opportunity for advancement and fulfillment of service terms among military personnel. Notably, bisexual veterans reported separating due to dissatisfaction with their job, and lesbian and bisexual women specifically reported separating due to dissatisfaction with promotions, pay, or benefits. While these data do not directly examine whether participants left the military because of their sexual orientation, this inequity warrants further examination to understand the underlying reasons for greater reports of non-routine separation among LGB veterans, given that previous research indicates that service members who identify as LGB have an elevated risk of experiencing discrimination, stalking, sexual harassment, and sexual assault while serving in the military [37–39]. Taken together, it is not necessarily surprising that LGB veterans were more likely to experience non-routine separations. Addressing these issues by increasing support and inclusion of LGB individuals in the military may have broader implications for the military. For example, it may increase retention of these qualified, well-trained service members, which would lead to better unit readiness and would help reduce costs associated with recruiting and training new service members.

We observed elevated frequencies of missed workdays due to illness or injury and medical separations among all LGB individuals, and post-separation unemployment due to disability among bisexual men and women compared with heterosexual individuals. This suggests that health concerns, which can lead to lost workdays, medical separation, and disability, may be higher among LGB versus heterosexual populations. While consistent with research in civilian and veteran populations that observed significant health disparities among LGB individuals [17, 40, 41], this finding is alarming given that active duty personnel have universal health care access and insurance coverage. Though the observed health concerns among LGB individuals could be partially related to other factors or mediators unrelated to military service that were not measured in the current study, such as childhood trauma or baseline differences in physical or mental health, this observation may also suggest an area of unaddressed disparity within the Military Health System (MHS) that could be attributable to a lack of knowledge and skill among military providers in providing culturally competent care for LGB individuals [42]. While pilot programs have been developed and implemented to promote cultural competence and educate health care providers about the unique health care issues of LGB service members [43, 44], currently there is no standardized MHS-wide cultural educational program for providers. Development and implementation of such programs, as well as increased training on contextually tailored or trauma-informed health care for all patients, both in the MHS and Veterans Health Administration, may help to increase cultural competence among health providers and in turn ameliorate adverse experiences among LGB service members and veterans.

We observed that bisexual service members were especially vulnerable to adverse outcomes during their military service and following separation, a finding consistent with research in civilian populations reporting poorer health and social outcomes among bisexual individuals compared with both heterosexual and gay or lesbian individuals [45, 46]. These differences may be partially attributable to minority stressors specific to bisexual individuals (e.g., stigma about bisexuality, double discrimination from both heterosexual and gay or lesbian individuals) [47].

While approximately half the observed associations did not differ by sex, there were some differences by sex in military and post-separation experiences. However, with few exceptions, these differences were fairly small in magnitude and the direction of the association was the same despite differences in effect size. Similarly, the observed associations did not differ by service status, with the exception of military support. Though there may be some minor differences in specific reasons for separation and morale, overall the findings indicate that LGB individuals, regardless of sex or service status, have an elevated risk for the adverse outcomes examined in this study. Thus, these findings continue to highlight the need for inclusion and cultural competence in the military environment with additional support and resources for all LGB individuals.

### Strengths and limitations

One of the main strengths of this study was the large proportion of the sample actively serving in the military, thus filling a significant gap in the literature where previous research had primarily been confined to veterans no longer serving in the military. To our knowledge, this is the largest sample of US service members and veterans representing all components, service branches, and ranks that assesses sexual orientation, thus making it possible to examine multiple small or underrepresented subgroups such as bisexual individuals, as well as differences across these subgroups by sex. In addition, the survey asked about numerous specific reasons for separation from service, including many not captured by administrative data.

There are notable limitations to the current study. While multiple response options for the sexual orientation question were available, additional identities (e.g., queer, pansexual, asexual, questioning) were not included. Thus, some participants may have endorsed an option inconsistent with their orientation or declined to respond because of limited options. These analyses were also limited by a lack of specific survey questions to assess gender identity. Participant sex was assessed using data from administrative records and likely reflects sex assigned at birth, thus precluding any investigation into how transgender or nonbinary gender identities affect military and separation experiences. Future studies using data from the Millennium Cohort Study plan to incorporate gender identity, as well as examine the role of other diversity-related disparities (e.g., differences among LGB individuals by race), to expand upon the current findings and examine military and separation experiences within intersecting identities. In addition, while we were able to assess a variety of military and service separation experiences, these experiences were not assessed in relation to the service member’s sexual orientation, so we cannot determine if the reported experiences were directly related to a participant’s sexual orientation (e.g., not feeling camaraderie due to discrimination). Given that sexual orientation remains a sensitive topic among military populations, even after the repeal of DADT, there may be an inherent response bias despite the use of confidential, secure, and self-administered surveys and assurances that survey data are not available to other military personnel or leadership. There may also be some level of bias in these data given the use self-reported measures with potentially long recall periods, such as missed days of work and reasons for separation. Finally, an adjusted comparison of pre- and post-DADT military experiences was not possible using these data due to the timing of participant enrollment with respect to the repeal of DADT in 2011. Within this population, all study participants served under DADT, less than 25% separated after its repeal, and none began their military service after its repeal. Thus, we do not have sufficient variation in our study population at this time to examine how reasons for separation differ across DADT time periods. However, temporal analyses of reasons for separation in relation to DADT using additional data as it becomes available in the future are of interest. Despite these limitations, the Millennium Cohort Program plans to enroll additional panels of service members in the post-DADT era. Future studies will be able to assess differences by sexual orientation across these eras.

## Conclusion

This analysis of a large sample of both US service members and veterans identified adverse military- and separation-specific experiences that disproportionately affected LGB individuals. A lack of inclusivity and cultural competence within the military environment may be negatively influencing how LGB service members perceive their military experiences and whether they are able to successfully complete their term of service. The identification of elevated risks for adverse military and post-separation experiences will be informative for military and Veterans Affairs leadership for the design and implementation of policies to address these inequities. Implementation of programs focused on promoting inclusion, training for cultural competence among health providers, and ensuring LGB individuals receive appropriate support from the military may be instrumental in improving overall satisfaction with military service and increasing readiness and retention.

## Data Availability

The datasets analyzed during the current study are not publicly available due institutional regulations protecting service member survey responses but are available from the corresponding author on reasonable request (may require data use agreements to be developed).

## Abbreviations

AOR: Adjusted Odds Ratio
CI: Confidence Interval
DADT: Don’t Ask, Don’t Tell
DMDC: Defense Manpower Data Center
LGB: Lesbian, Gay, or Bisexual
MHS: Military Health System
US: United States
